# Dichotic listening performance and interhemispheric integration after administration of hydrocortisone

**DOI:** 10.1038/s41598-021-00896-1

**Published:** 2021-11-03

**Authors:** Gesa Berretz, Julian Packheiser, Oliver Höffken, Oliver T. Wolf, Sebastian Ocklenburg

**Affiliations:** 1 Department of Biopsychology, Faculty of Psychology, Institute of Cognitive Neuroscience, Ruhr University Bochum, Universitätsstraße 150, IB 6-109, Bochum, Germany; 2 Department of Neurology, BG-University Clinic Bergmannsheil, Ruhr University Bochum, Bochum, Germany; 3 Department of Cognitive Psychology, Faculty of Psychology, Institute of Cognitive Neuroscience, Ruhr University Bochum, Bochum, Germany; 4 Netherlands Institute for Neuroscience, Social Brain Lab, Amsterdam, The Netherlands; 5 Department of Psychology, Medical School Hamburg, Hamburg, Germany

**Keywords:** Human behaviour, Psychology, Endocrinology, Neuroscience, Stress and resilience

## Abstract

Chronic stress has been shown to have long-term effects on functional hemispheric asymmetries in both humans and non-human species. The short-term effects of acute stress exposure on functional hemispheric asymmetries are less well investigated. It has been suggested that acute stress can affect functional hemispheric asymmetries by modulating inhibitory function of the corpus callosum, the white matter pathway that connects the two hemispheres. On the molecular level, this modulation may be caused by a stress-related increase in cortisol, a major stress hormone. Therefore, it was the aim of the present study to investigate the acute effects of cortisol on functional hemispheric asymmetries. Overall, 60 participants were tested after administration of 20 mg hydrocortisone or a placebo tablet in a cross-over design. Both times, a verbal and an emotional dichotic listening task to assess language and emotional lateralization, as well as a Banich–Belger task to assess interhemispheric integration were applied. Lateralization quotients were determined for both reaction times and correctly identified syllables in both dichotic listening tasks. In the Banich–Belger task, across-field advantages were determined to quantify interhemispheric integration. While we could replicate previously reported findings for these tasks in the placebo session, we could not detect any differences in asymmetry between hydrocortisone and placebo treatment. This partially corroborates the results of a previous study we performed using social stress to induce cortisol increases. This suggests that an increase in cortisol does not influence dichotic listening performance on a behavioral level. As other studies reported an effect of stress hormones on functional hemispheric asymmetries on a neuro-functional level, future research using neuronal imaging methods would be helpful in the characterization of the relation of hemispheric asymmetries and stress hormones.

## Introduction

Hemispheric asymmetries constitute a basic organizational principle of the vertebrate brain^1^. On the functional level, asymmetries emerge through dominance of one hemisphere for processing in a given task; many cortical processes like language perception or hand motor control are lateralized to one hemisphere^2,3^. While networks in both hemispheres contribute to task processing, each hemisphere is specialized for different aspects of the task^4^.

A possible mechanism underlying the emergence of functional hemispheric asymmetries (FHAs) is inhibition through the corpus callosum^5,6^: glutamatergic fibers in the corpus callosum synapse on GABAergic interneurons^7^. Thus, activation of the dominant hemisphere can lead to inhibition of the non-dominant hemisphere during task processing. This inhibition can be modulated via hormonal influences^8^. Several psychiatric and neurodevelopmental disorders have been associated with changes in structural and functional hemispheric asymmetries^9–11^. Importantly, stress has been suggested to be a major influence factor in the pathogenesis of almost all of these disorders and most of them have been related to changes in basal or stress induced cortisol concentrations^12^. Cortisol constitutes one of the major stress hormones in the human body^13^. There are two systems that respond to acute stress: The sympathetic nervous system constitutes the faster one, triggering release of adrenaline and noradrenaline from the adrenal medulla^14^. As an indirect measure of sympathetic nervous system activity, alpha amylase in saliva can be used^15^. The slower-acting Hypothalamus–Pituitary–Adrenal (HPA) axis triggers secretion of corticotrophin-releasing hormone from the hypothalamus. This in turn stimulates the release of adrenocorticotropic hormone from the anterior pituitary, which leads to release of cortisol from the cortex of the adrenal medulla^16^. Cortisol affects cortical networks by binding to glucocorticoid (GR) and mineralocorticoid (MR) receptors^17^.

While changes in cortisol levels are linked with neurodevelopmental and mental disorders, effects of acute stress and the accompanying stress hormones on FHAs are not well understood as only a few selective studies investigated this association. A study by Brüne et al.^18^ for example reported faster responses to negative stimuli in the left visual half field. In contrast, faster responses to positive stimuli were found after stimuli were presented in the right visual half field in the stress condition. Similarly, a recent study by Stanković & Nešić^19^ found that after watching a stressful movie clip, left-hemispheric dominance for emotional face perception increased. These findings suggest that acute stress can affect FHAs.

At least two possible mechanisms could mediate the influence of stress on FHAs: in the affective model, it is assumed that the negative emotions associated with acute stress selectively prime the right hemisphere as the right hemisphere is proposed to be dominant for emotion processing^20^. In the psychoneuroendocrine model^21^, stress-related cortisol release is postulated to affect FHAs by modulating glutamatergic and GABAergic neurotransmission via the corpus callosum. While progesterone has been suggested to lead to a decoupling of the hemispheres^8^, cortisol enhances glutamatergic transmission and can therefore enhance FHAs^22,23^. In a recent study, we found that acute stress and the associated increase in cortisol did not influence dichotic listening performance but facilitated interhemispheric integration of information^24^. As we employed the Trier Social Stress Test^25^ for stress induction in this previous study, not only cortisol but also psychological stress measures and markers of SNS activity were increased.

Based on these findings, it was the aim of the present study to assess the influence of cortisol on FHAs. To disentangle the effects of endocrinological and affective parameters, we repeated the experiment conducted by Berretz et al.^24^ with a pharmacological intervention instead of a psycho-social stress induction. As stress induction not only increases cortisol levels and levels of other stress modulators but also evokes an affective component that engages a multitude of cognitive capacities^26^, it is necessary to influence cortisol levels through pharmacological administration to characterize its specific and unique effects on the central nervous system^27^.

Participants performed three different tasks assessing FHAs and interhemispheric integration. Stimuli in the verbal dichotic listening task consist of two syllables that are each presented to the left or right ear simultaneously^28^. Participants typically report more syllables presented to the right ear which is called the right-ear advantage^29^. This advantage can be found for auditory as well as verbal imagery material^30,31^ and reflects left hemispheric language lateralization^32,33^. In the emotional version of the dichotic listening task, the same word is presented to both ears but in different emotional intonations on the left and on the right. Participants report more syllables that were presented to the left ear reflecting right hemispheric emotion lateralization^34^.

The Banich–Belger task assesses interhemispheric integration^35^. Participants are asked to compare stimuli in the right and left visual half field. Compared to the physical-matching condition, participants typically show better performance in the more taxing name-matching condition when stimuli are displayed across both visual fields. Thus, the task measures integration of information across the corpus callosum^36^.

We expect that participants will display a typical right-ear advantage in the verbal dichotic listening task and a left-ear advantage in the emotional dichotic listening task indicating right-hemispheric processing and left hemispheric processing, respectively. In the Banich–Belger task, we expect participants to profit from interhemispheric integration leading to shorter reaction times during across field trials in the name-matching condition. In line with the psychoneuroendocrine model, we expect stronger FHAs in the dichotic listening tasks as well as a positive effect on interhemispheric integration in the Banich–Belger task.

## Methods

### Participants

We recruited all participants at the Ruhr University Bochum, Germany. The cohort consisted of 60 participants (31 males and 29 females), with an age range from 18 to 34 years (mean age = 23.62 years, SD = 3.85). The sample size was determined beforehand with a power analysis using g*Power^37^ with an α-error probability of 0.05 and a power of 0.95. As the main effect of interest in the present study was the interaction between treatment and laterality indices in an ANOVA, we estimated the effect size to be small (partial η^2^ = 0.07) based on the data by Brüne et al.^18^. Six participants were left-handed as they displayed a negative handedness LQ measured with the Edinburgh Handedness Inventory^38^. All other participants were right-handed as they had positive handedness LQs (M = 71.83, SD = 52.87, min = -100, max = 100). We chose to include left-handers in accordance with recent recommendations for studies on hemispheric asymmetries^39^. All participants were healthy without any history of mental or neurological disorders. Exclusion criteria included intake of medication, hormonal contraceptives or drugs. To control for possible influences on cortisol levels, all participants had a body mass index between 18.5 and 28 kg/m^2^ and did not perform shiftwork^40^. Participants’ hearing abilities were screened with an audiometer (MAICO Diagnostics GmbH, Berlin, Germany) to assure no differences in hearing ability between both ears (cutoff: 15 dB difference between ears). The local ethics committee of the Faculty of Medicine at the Ruhr University Bochum approved the study and participants were treated in accordance with the Declaration of Helsinki. Before participation, written informed consent from all participants was obtained.

### Procedure

Participants were tested in two sessions at the Ruhr University Bochum taking place between 2 and 8 pm. Female participants were only tested in the early follicular phase of their cycle to control for influences of fluctuations of hormone levels on FHAs^8^. After completion of subjective stress and cortisol measurements at baseline, participants were given either two tablets of 10 mg hydrocortisone each or a placebo. The dosage of 20 mg has been used successfully in previous studies by our group (e.g.^41^). Hydrocortisone and placebo condition were pseudorandomized between participants. Subsequently, participants waited 40 min before proceeding with the experiment as the hydrocortisone tablet need this time to be dissolved and absorbed into the body.

**Figure 1:**
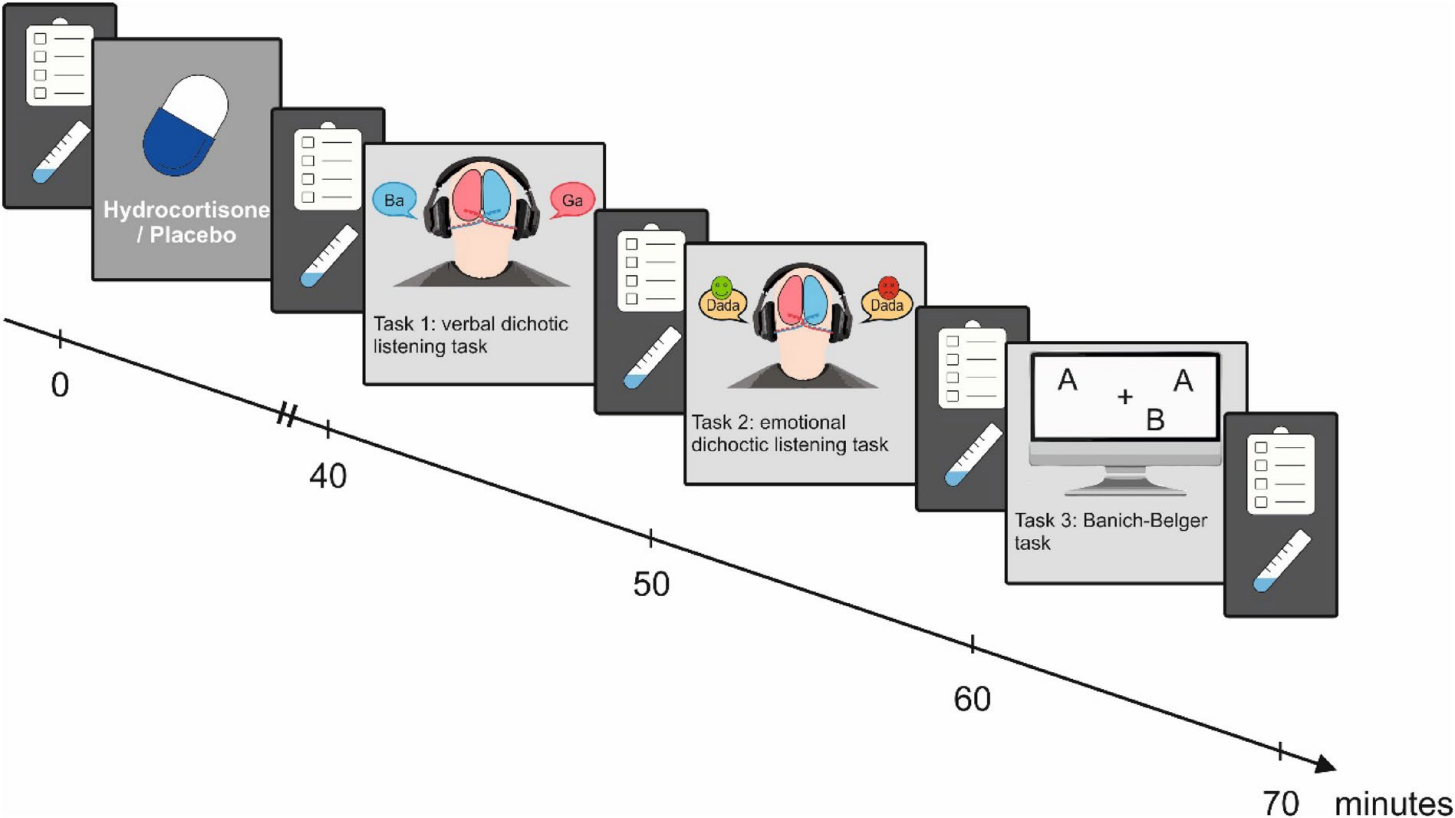
Experimental design. After administration of hydrocortisone or placebo, the participant is tested using verbal dichotic listening, emotional dichotic listening and the Banich–Belger task. Before hydrocortisone administration and after each section of the experiment, cortisol and affect are assessed.

After this waiting period as well as between all later tasks, salivary samples were collected using Salivette sampling devices (Sarstedt AG, Germany). With each assessment, we also assessed the mood of the participants (see Fig. 1) using the Subjective Experiences Rating Scale (SERS;^42^) as well as a set of visual analog scales that measure subjective perception of stress (VAS;^43^).

### Endocrinological measurements

To assess the effectiveness of hydrocortisone administration, salivary cortisol and salivary alpha-amylase activity were measured at five time points across the experiment. Salivary alpha-amylase was used as a marker for sympathetic nervous system activity. Samples were stored at − 20 °C until analysis. Saliva samples were first 20 × diluted. Salivary cortisol was analyzed on a Synergy2 plate reader (Biotek, USA) using a commercial enzyme-linked immunosorbent assay (ELISAs; free cortisol in saliva; IBL/Tecan, Hamburg) according to the manufacturer’s instructions. Intra- and interassay variability of the assay was less than 7%. A colorimetric test using 2-chloro-4-nitrophenyl-α-maltotriosoide (CNP-G3) as a substrate reagent was applied to measure salivary alpha-amylase (sAA) activity as described elsewhere^44^ and had an intra- and interassay variability of less than 4% and 5%, respectively.

### Experimental paradigms

In this study, participants completed three different tasks. The order in which the paradigms were conducted was pseudo-randomized within each session and across participants. All paradigms were programmed and presented using the software Presentation (Neurobehavioral Systems, Inc., Albany, USA).

#### Banich–Belger task

The Banich–Belger task measures interhemispheric information transfer^36^. To ensure that participants’ eyes stayed at a fixed distance of 50 cm from the screen, a headrest was used and participants were instructed to keep their heads on the headrest. In the center of the screen, a fixation cross was presented and participants were instructed to fixate it at all times during the experiment. Two different letters were presented above the fixation cross and a third letter was presented below it. The lower letter was presented either in the right or in the left visual field. The stimuli consisted of the letters A, B, E, G, H, Q, R, and T. The two letters in the upper part of the screen (probe letters) were presented at 2.8° of visual angle to the left or right side and 1.4° visual angle above the fixation cross. The letter in the lower half of the screen (target letter) was presented at 1.4° visual angle to the left or the right side and 1.4° visual angle below the fixation cross. Two different conditions that varied in difficulty were administered as part of the task: In the easier physical-matching condition, all letters were upper case; in the more difficult name-matching condition, the lower letter was a lowercase letter. For both conditions, participants have to press a button to indicate, whether the lower letter was identical with either one of the upper letters or not. Each trial began with the presentation of the fixation cross for 200 ms. Subsequently, a stimulus was presented for 200 ms. The intertrial interval was jittered to avoid habituation effects and had a length between 500 and 2000 ms duration. Before the task started, participants had to conclude 14 training trials with each hand. These training trials were excluded from later analyses. Within each block, half of the trials were so-called match-trials. In this type of trial, the lower letter did coincide with either one of the upper letters. The other half of trials were mismatch trials, in which the lower letter was not identical with one of the upper letters. Half of the match trials were across-field match trials in which target letter and probe were in two different visual fields. The other half of match trails were within-field matches in which target letter and probe were in the same visual field. Within both types of match trials, the bottom letter appeared with the same frequency in the right visual field (RVF) and left visual field (LVF). In total, participants completed 256 trials in four blocks. Each block contained 64 trials (32 match and 32 mismatch trials). The first two blocks belonged to the physical-matching condition and the last two blocks to the name-matching condition. Participants changed response hand after each block. The task took about 15 min to complete.

#### Verbal dichotic listening task

As stimuli, different syllable pairs were constructed from two different consonant–vowel pairs with one syllable presented to each ear spoken in a male voice (ba, da, ga, ka, pa, ta;^45^). Participants were asked to press one of six keys on a customized response pad corresponding to the syllable they had perceived best. Participants mostly kept their hand in a neutral position from which out they moved their whole hand to the response key mitigating possible effects on response time. Mean duration of stimulus presentation was 350 ms at a volume of 80 dB. The inter-stimulus interval varied between 500 and 1000 ms. After 12 training trials, participants completed 144 trials in four blocks of 36 trials each. Within each block, all 36 possible combinations of syllable pairs were presented. Participants were instructed to change the hand with which they responded after each block. This task had a duration of 10 min.

#### Emotional dichotic listen task

As stimuli, the word “dada” spoken in five different emotional intonations spoken in a female voice (happy, sad, neutral, angry, surprised;^46^) was used. Participants were asked to press one of five keys corresponding to the emotion they had perceived best. The inter-stimulus interval varied between 500 and 1000 ms. After 12 training trials, participants completed a total of 100 trials in four blocks of 25 trials with all 25 possible combinations of emotion pairs. Again, participants were instructed to change the hand with which they responded after each block. This task also had a duration of 10 min.

### Statistical analysis

For all analyses, we used only reaction times and accuracy of valid trials. Trials were classified as valid if participants correctly indicated if the lower letter was identical with one of the upper letters in the Banich–Belger task. In the dichotic listening tasks, responses were classified as valid if participants responded to either the left or right stimulus.

For the Banich–Belger task, we calculated mean reaction times and mean number of correct responses for each visual half field in each condition for each participant. For both dichotic listening tasks, we calculated mean reaction times and mean number of correct responses for each side of stimulus presentation for each participant.

We calculated lateralization quotients (LQs) in both dichotic listening tasks for mean reaction times and numbers of correct responses following the formula^38,47^:

$${\text{LQ}} = \left[ {\left( {{\text{right}}{-}{\text{left}}} \right){/}\left( {{\text{right}} + {\text{left}}} \right)} \right]*100$$

We calculated the Across Field Advantage (AFA) in the Banich–Belger task by subtracting mean reaction times on across-field trials from mean reaction times on within-field trials for both task conditions as well as total AFAs by computing an average across name- and physical-matching condition.

We calculated repeated measures ANOVA (rmANOVA) with the factors treatment, condition and visual field in the Banich–Belger task and the factors treatment and ear in the dichotic listening tasks.

The analysis was repeated using a Bayesian rmANOVA to further investigate whether there was indeed no difference between hydrocortisone and placebo treatment. Instead of p-values and effect sizes, we here report the BF_M_. The BF_M_ illustrates the changes from prior to the posterior odds under a certain model in the rmANOVA. For t-tests and correlation analyses, the BF_10_ factor was used. For both the BF_M_ and the BF_10_, a value of greater than 1 indicates stronger evidence for the alternative hypothesis whereas a value of lower than 1 indicates stronger evidence in favor of the null hypothesis.

#### Cortisol response

To evaluate the effectiveness of the hydrocortisone administration, we computed separate rmANOVAs with the factors treatment (hydrocortisone vs placebo) and the measurement time points (T1–T5) using cortisol, alpha amylase as well as SERS and VAS scores as dependent variable. As the original values were not normally distributed, we log-transformed cortisol and salivary alpha amylase values. For the cortisol and sAA data, we calculated the area under the curve with respect to baseline (AUC_i_) reflecting changes in hormone levels following the method detailed in Pruessner et al.^48^. We calculated subjective stress reactivity by subtracting SERS scores at the first measurement time point from the second time point. We excluded two participants of whom no cortisol data was available since participants did not provide enough saliva for the analysis. Thus, all following analyses were calculated with the remaining 58 participants.

## Results

### Hydrocortisone administration

**Figure 2:**
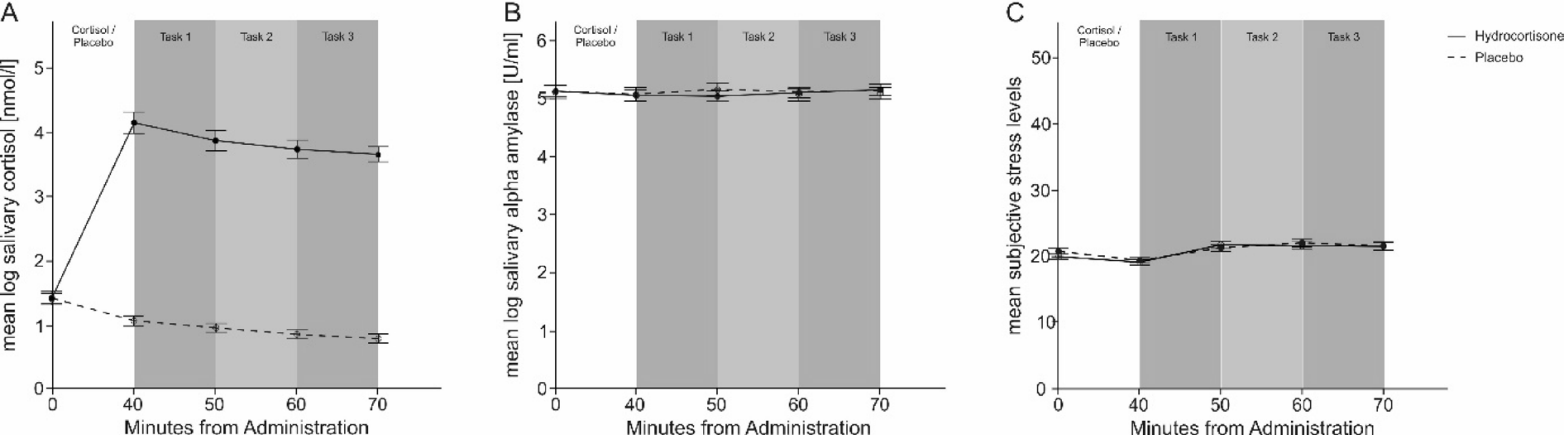
Physiological and subjective reactions during hydrocortisone and placebo treatment. Error bars represent 1 ± SEM from the mean. (**A**) Mean cortisol responses in relation to measurement time point with logarithmized data. Cortisol levels were increased in the hydrocortisone group from the second measurement time point onwards. (**B**) Mean salivary alpha amylase response in relation to measurement time point with logarithmized data. No changes in sAA levels were observed between the hydrocortisone and control group across the experiment. (**C**) Mean subjective stress responses measured by SERS in relation to measurement time point. No differences in SERS levels were observed between the hydrocortisone and control group across the experiment.

The rmANOVA comparing between hydrocortisone and placebo treatment demonstrated a significant main effect of treatment (F_(1,57)_ = 336.66, *p* < .001, η_p_^2^ = 0.86) and measurement time point (F_(4,228)_ = 98.33, *p* < .001, η_p_^2^ = 0.63). Furthermore, there was an interaction effect of both factors (F_(4,228)_ = 200.42, *p* < .001, η_p_^2^ = 0.78, see Fig. 2A). Post-hoc tests using Bonferroni correction revealed that salivary cortisol was increased for the second, third, fourth and fifth measurement time point with respect to the first time point during hydrocortisone treatment (all *p*s < .001). Using sAA as dependent variable, we found neither a significant main effect nor a significant interaction of treatment and measurement time point (all *p*s > .52, see Fig. 2B).

The rmANOVA comparing the SERS and VAS scores (see Fig. 2C) during hydrocortisone and placebo treatment exhibited a significant main effect of measurement time point (F_(4,228)_ = 17.19, *p* < .001, η_p_^2^ = 0.23). Post hoc tests revealed that SERS scores at the second measurement time point were decreased compared to all other scores (all *p*s < .05). For VAS scores, there was also a significant main effect of measurement time point (F_(4,228)_ = 73.99, *p* < .001, η_p_^2^ = 0.57). Post-hoc tests revealed that the first two measurement time points demonstrated lower scores compared to the other time points (all *p*s < .001). These results demonstrate that administration of hydrocortisone selectively elevated cortisol levels while leaving sAA and subjective stress feelings unaffected.

### Banich–Belger task

#### Correct responses

**Figure 3:**
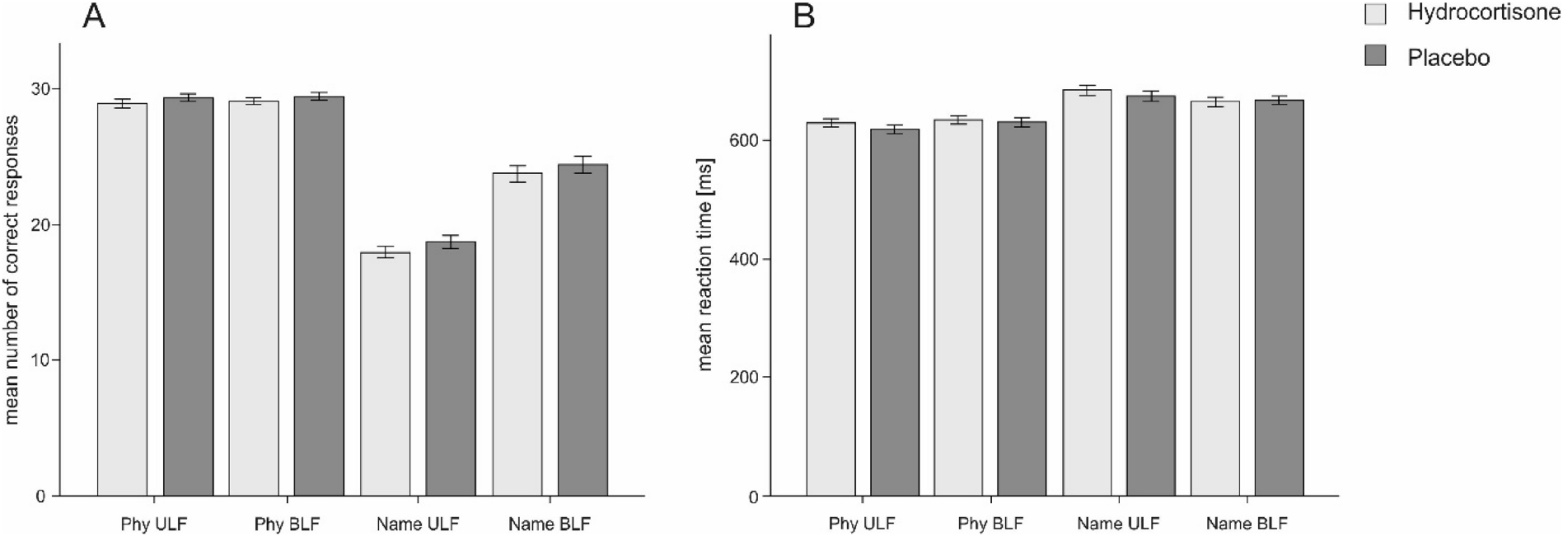
Mean number of correct responses (**A**) and mean reaction times (**B**) for each condition and visual field in the Banich–Belger task. Error bars represent ± 1 SEM. Physical and name matching conditions and unilateral and bilateral field are depicted.

For the analysis of the Banich–Belger task, we computed a 2 × 2 × 2 repeated measures ANOVAs with the factors treatment (hydrocortisone vs placebo), condition (physical- vs name-matching) and visual field (across vs within) for the number of correct responses (see Supplementary Table 1). The ANOVA revealed a significant main effect of condition (F_(1,57)_ = 512.03, *p* < .001, η_p_^2^ = 0.90) and visual field (F_(1,57)_ = 217.78, *p* < .001, η_p_^2^ = 0.79). There was also an interaction effect between the factors condition and visual field (F_1,57)_ = 203.02, *p* < .001, η_p_^2^ = 0.78). Post-hoc tests using Bonferroni correction showed that there was a significant decrease in correct responses in the name matching condition when performing within-field trials (*p* < .001). No main effect of treatment nor any interactions involving treatment as a factor was observed (all *p*s > .160, see Fig. 3A). Reliability coefficients for the task can be found in Supplementary Table 4.

We found strong evidence for the alternative hypothesis for the model containing the main effect of condition (BF_M_ = 29.15). No model containing the factor treatment provided more than anecdotal evidence for the alternative hypothesis (all BF_M_s < 2.58) which can be considered negligible. Rather, all models containing the main effect of treatment and any interaction with this factor provided strong evidence in favor of the null hypothesis (all BF_M_s < 0.46).

#### Reaction times

The rmANOVA was repeated using reaction times as dependent variable (see Supplementary Table 1). As for correct responses, we found a significant main effect of condition (F_(1,57)_ = 36.21, *p* < .001, η_p_^2^ = 0.39) as well as a significant interaction between condition and visual field (F_(1,57)_ = 6.73, *p* < .05, η_p_^2^ = 0.11). Bonferroni corrected post-hoc tests demonstrated faster reaction times on within-field trials in the physical-matching condition and faster reaction times on between across-field trials in the name-matching condition (*p* < .001). There were no other significant effects (*p*s > .208, see Fig. 3B).

The Bayesian rmANOVA found strong evidence supporting the alternative hypothesis for the model containing the main effect of condition (BF_M_ = 36.71). No model containing the factor treatment provided more than anecdotal evidence for the alternative hypothesis (all BF_M_s < 1.68).

#### Across-field advantage

We computed a t-test for dependent samples between total across-field advantages (AFAs) of hydrocortisone and placebo treatment. There was no significant difference between hydrocortisone and placebo treatment for AFA in the physical matching condition (t_(57)_ = 0.61, *p* = .542), in the name matching condition (t_(57)_ = 1.10, *p* = .275) nor in total AFAs (t_(57)_ = 1.27, *p* = .208). A Bayesian t-test revealed anecdotal evidence in favor of the null hypothesis (BF_10_ = 0.364).

#### Correlation with stress markers

To investigate a possible association between test performance in the Banich–Belger task and stress-related variables, the analysis was complemented by Bayesian correlations. For this purpose, we calculated Pearson correlations between cortisol, sAA as well as the SERS scores and AFAs.

We found no significant correlation during hydrocortisone treatment between AFAs and cortisol (r_(58)_ = 0.01, *p* = .933), sAA (r_(58)_ = 0.07, *p* = .612) and SERS scores (r_(58)_ = 0.05, *p* = .703). There was substantial evidence for the null hypothesis during hydrocortisone treatment for cortisol (BF_10_ = 0.16), sAA (BF_10_ = 0.18) and SERS Scores (BF_10_ = 0.17).

We also found no significant associations during placebo treatment between AFAs and cortisol (r_(58)_ = -0.02, *p* = .866), sAA (r_(58)_ = -0.20, *p* = .124) and SERS scores (r_(58)_ = -0.10, *p* = .430). Again, there was substantial evidence favoring the null hypothesis during placebo treatment for cortisol (BF_10_ = 0.17), sAA (BF_10_ = 0.53) and SERS Scores (BF_10_ = 0.22).

Finally, we investigated the interaction effects of cortisol and sAA on interhemispheric integration. To this end, we calculated a multiple linear regression analysis with log-transformed cortisol and sAA AUC_i_ values as predictors and AFAs of reaction times as dependent variable for hydrocortisone treatment. The model failed to reach significance (R^2^ = 0.01, F_(2,55)_ = 0.18, *p* = .838).

### Verbal dichotic listening task

#### Correct responses

**Figure 4:**
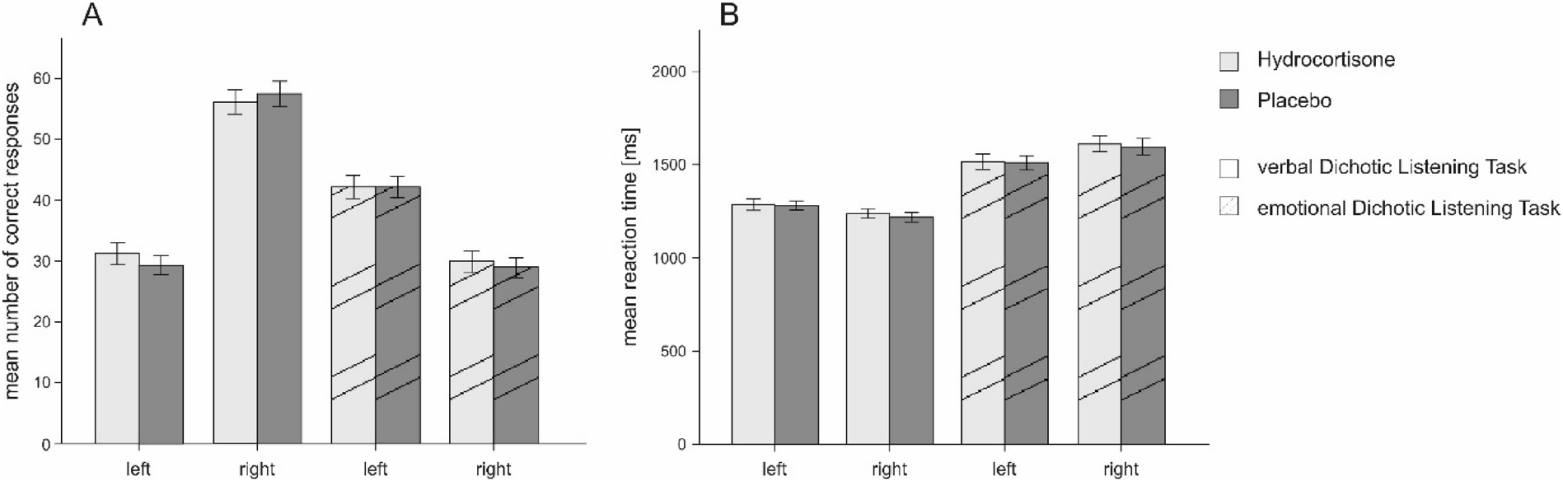
Mean number of correct responses per ear (**A**) and mean reaction times in ms (**B**). Solid bars indicate the verbal dichotic listening task; striped bars indicate the emotional dichotic listening task. Participants reported more stimuli presented and were faster in responding to stimuli on the right than on the left side indicating a left hemispheric lateralization for language for the verbal dichotic listening task. Participants reported more stimuli presented and were faster in responding to stimuli on the left than on the right side indicating a right hemispheric lateralization for emotion processing for the emotional dichotic listening task. Error bars represent ± 1 SEM.

A 2 × 2 rmANOVA with the factors treatment (hydrocortisone vs placebo) and ear (left ear vs. right ear) revealed a significant main effect of ear (F_(1,56)_ = 64.10, *p* < .001, η_p_^2^ = 0.53; see Supplementary Table 2). Here, more correct responses were reported on the right ear (see Fig. 4A). We complemented this analysis by a Bayesian ANOVA and found strong evidence favoring the alternative hypothesis for the model containing the main effect of ear (BF_M_ = 22.88). All models containing the factor treatment provided substantial evidence in favor of the null hypothesis (all BF_M_s < 0.53). Reliability coefficients for the task can be found in Supplementary Table 5.

Comparing hydrocortisone and placebo treatment using a dependent sample t-test for the LQ of number of correct responses did not reveal significant results (t_(56)_ = 1.07, *p* = .290, see Fig. 4A). These findings were supported by Bayesian t-tests demonstrating substantial evidence favoring the null hypothesis for correct responses (BF_10_ = 0.23). There was furthermore no significant association between LQs and cortisol (r = 0.06, *p* = .681, see Fig. 4), sAA (r = − 0.08, *p* = .540) or SERS scores (r = 0.08, *p* = .546) in the hydrocortisone treatment. Bayesian correlation analyses supported these results as they provided evidence in favor of the null hypothesis for all variables (cortisol: BF_10_ = 0.18; sAA: BF_10_ = 0.20; SERS score: BF_10_ = 0.19). For the placebo treatment, we found comparable results since no association between the LQs for correct responses and cortisol (r = − 0.03, *p* = .844), sAA (r = 0.10, *p* = .480) or SERS scores (r = − 0.01, *p* = .970) could be detected. Again, Bayesian correlation analyses favored the null hypothesis for all variables (cortisol: BF_10_ = 0.33; sAA: BF_10_ = 0.17; SERS score: BF_10_ = 0.31).

#### Reaction times

Participants exhibited a negative LQ for reaction times indicating faster reaction times to stimuli that were presented on the right ear (see Fig. 4B). The 2 × 2 rmANOVA containing the factors experimental treatment and side showed a significant main effect of ear (F_(1,56)_ = 20.62, *p* < .001, η_p_^2^ = 0.27; see Supplementary Table 2). Here, reaction times were faster for stimuli presented on the right ear as well. Complementing these results with a Bayesian ANOVA, we found strong evidence for the alternative hypothesis for the model containing the main effect of side (BF_M_ = 15.28). All models comprising the factor treatment provided substantial evidence in favor of the null hypothesis (all BF_M_s < 0.73).

**Figure 5:**
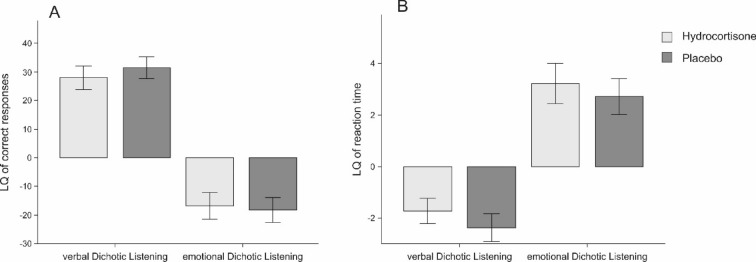
LQs for the verbal and emotional dichotic listening task for number of correct (**A**) and responses mean reaction times (**B**). Error bars represent ± 1 SEM.

LQs of mean reaction times were compared using a dependent sample t-test between the hydrocortisone and placebo treatment. The test did not reach significance (t_(56)_ = 1.05, *p* = .296, see Fig. 4B). This finding was supported by a Bayesian t-test that revealed substantial evidence favoring the null hypothesis for reaction times (BF_10_ = 0.26). We further investigated whether individual cortisol, sAA and subjective stress levels were associated with reaction time LQs in the verbal dichotic listening task. We found no significant association with LQs for cortisol (r = 0.02, *p* = .861, see Fig. 5), sAA (r = − 0.12, *p* = .362) or SERS scores (r = − 0.18, *p* = .173) during hydrocortisone treatment. Bayesian correlations favored the null hypothesis for all stress-related variables (cortisol: BF_10_ = 0.17; sAA: BF_10_ = 0.25; SERS score: BF_10_ = 0.40). For the placebo treatment, there was also no significant association between reaction times LQs and cortisol (r = 0.24, *p* = .075). Levels of sAA (r = 0.33, *p* < .05) and SERS scores (r = − 0.29, *p* = .026) were however significantly correlated to reaction times LQs. Higher LQs were therefore accompanied by higher sAA and lower SERS scores. Bayesian correlation analyses favored the null hypothesis for cortisol (cortisol: BF_10_ = 0.77). In the case of sAA (BF_10_ = 3.86) and SERS scores (BF_10_ = 1.85), these analyses however favored the alternative hypothesis.

### Emotional dichotic listening task

#### Correct responses

In the emotional dichotic listening task, participants exhibited an overall negative LQ for number of correct responses indicating that they perceived a higher number of syllables on the left ear. The 2 × 2 rmANOVA containing the factors treatment (hydrocortisone vs placebo) and ear (left vs right) revealed a significant main effect of ear (F_(1,57)_ = 16.51, *p* < .001, η_p_^2^ = 0.23, see Supplementary Table 3) with more correct responses being reported on the left ear (see Fig. 4A). We repeated this analysis using a Bayesian ANOVA and found strong evidence for the alternative hypothesis for the model containing the main effect of side (BF_M_ = 21.54). All models containing the factor treatment provided substantial evidence for the null hypothesis (all BF_M_s < 0.60). Reliability coefficients for the task can be found in Supplementary Table 6.

The LQs for the number of correct responses were compared between hydrocortisone and placebo treatment using a dependent sample t-test which did not reach significance (t_(57)_ = 0.67, *p* = .506, see Fig. 5A). These results were corroborated by a Bayesian t-test revealing strong evidence favoring the null hypothesis (BF_10_ = 0.18). We furthermore found no significant correlation between LQs and cortisol (r = 0.22, *p* = .102) and SERS scores (r = 0.14, *p* = .276) during hydrocortisone treatment. There was however a significant negative correlation with sAA levels (r = − 0.30, *p* < .05). Here, higher levels of cortisol were associated with more negative LQs. Bayesian correlation analyses favored the null hypothesis model for cortisol and subjective stress but favored the alternative hypothesis model for sAA (cortisol: BF_10_ = 0.60; sAA: BF_10_ = 2.18; SERS score: BF_10_ = 0.29). During placebo treatment, we found comparable results since no association between correct responses LQs and cortisol (r = 0.21, *p* = .103), sAA (r = − 0.05, *p* = .728) and SERS scores (r = − 0.21, *p* = .101) could be detected. As before, Bayesian correlation analyses favored the null hypothesis for all stress-related variables (cortisol: BF_10_ = 0.59; sAA: BF_10_ = 0.17; SERS score: BF_10_ = 0.60).

#### Reaction times

For reaction times, participants demonstrated a positive LQ suggesting faster reactions to stimuli presented on the left ear (see Fig. 4B). The 2 × 2 rmANOVA with the factors treatment (hydrocortisone vs placebo) and side (left vs right) revealed a significant main effect of side (F_(1,56)_ = 21.56, *p* < .001, η_p_^2^ = 0.28, see Supplementary Table 3). Here, stimuli presented on the left ear elicited faster reaction times as well. Complementing these findings with a Bayesian ANOVA, we found strong evidence favoring the alternative hypothesis for the model containing the main effect of side (BF_M_ = 18.60). All models containing the factor treatment provided substantial evidence for the null hypothesis (all BF_M_s < 0.64).

The reaction times were furthermore compared between hydrocortisone and placebo treatment using a dependent sample t-test. The test did not reach significance (t_(56)_ = 0.50, *p* = .621, see Fig. 5B). These results were supported by a Bayesian t-test demonstrating strong evidence in favor of the null hypothesis model (BF_10_ = 0.16). We then investigated whether individual cortisol, sAA and SERS scores were associated with LQs in the verbal dichotic listening task. For reaction times, there was no significant correlation between LQs and cortisol (r = − 0.18, *p* = .185), sAA (r = 0.16, *p* = .246) or SERS scores (r = − 0.04, *p* = .783) during hydrocortisone treatment. Bayesian correlation analyses for the association between cortisol, sAA and SERS scores supported the null hypothesis model (cortisol: BF_10_ = 0.39; sAA: BF_10_ = 0.32; SERS score: BF_10_ = 0.17). During placebo treatment, we found no association between correct responses LQs and cortisol (r = 0.00, *p* = .983), sAA (r = − 0.01, *p* = .926), and SERS scores (r = 0.05, *p* = .725). Bayesian correlation analyses for these associations indicated in favor of the null hypothesis for all stress-related variables (cortisol: BF_10_ = 0.16; sAA: BF_10_ = 0.16; SERS score: BF_10_ = 0.17).

## Discussion

In the present study, we investigated the influence of pharmacological administration of hydrocortisone on interhemispheric integration and FHAs. Administration of hydrocortisone lead to a robust increase in salivary cortisol but to no changes in sAA or subjective stress which is in line with previous work^49,50^. For all tasks, we could replicate the expected results during the placebo condition: participants displayed a right ear advantage in the verbal dichotic listening task^28^. This indicated typical left lateralization of language perception^29,51^. We also found a left ear advantage in the emotional dichotic listening task reflecting right-hemispheric dominance for processing of emotions^46,52^. In the Banich–Belger task, participants showed shorter reaction times in the name matching condition on across trials indicating an advantage of information integration across both hemispheres in more difficult tasks^36^. Neither in the Banich–Belger task nor in the two dichotic listening tasks, could we find any differences between the hydrocortisone and placebo treatment regarding changes in functional hemispheric asymmetries. Moreover, we could not detect any association between cortisol and subjective stress ratings and asymmetry indices in the hydrocortisone treatment. However, there was a significant association between sAA and the LQ for correct responses in the emotional dichotic listening task. Moreover, there was an association between sAA and subjective stress and LQ for reaction times in the verbal dichotic listening task during placebo treatment. This indicated that lower subjective levels of stress and higher sympathetic activity were associated with a higher LQ of reaction times and thus slower performance of the left hemisphere. Using Bayesian statistics, we confirmed these results, as there was substantial evidence in favor of the null hypothesis with regard to the influence of cortisol on hemispheric asymmetries.

We partly confirmed our initial hypotheses since there was indeed no effect of cortisol increases on FHAs as measured via an emotional and verbal dichotic listening task in line with the findings of Berretz et al.^24^. Previous studies by Brüne et al.^18^ and Stanković & Nešić^19^ found effects of stress on lateralized face perception. However, these findings are not necessarily in opposition to our results. It has to be noted that these studies chose different stimulus materials and methods of stress induction, which somewhat limits the comparability with the current study. Brüne et al.^18^ used emotional faces as stimuli and found faster reaction times under stress in the left visual field (right hemispheric advantage). Stanković & Nešić^19^ found that watching a control movie clip led to an equalization of FHAs compared to the left visual field advantage for emotional faces detected at baseline. In the stress condition, they however found that the right visual field gained an advantage in the perception of emotional faces. While both studies found a group level effect, neither study investigated the direct relationship between cortisol levels and FHAs. As our study focused specifically on the influence of cortisol on FHAs, the psychoneuroendocrine model, which suggests that cortisol alters FHAs through interhemispheric inhibition across the corpus callosum, cannot be supported on the basis of our data. Thus, taken together these findings and the findings of the present study suggest that it may be possible that not all forms of FHAs are amenable to the influence of stress and stress hormones. More research in a wider amount of different forms of functional hemispheric asymmetries is therefore needed to understand this interesting pattern of results.

We found no relationship between cortisol or sAA levels and interhemispheric integration. For sAA, this result is unsurprising since our study did not modulate sympathetic stress responses. Thus, sAA levels were stable throughout the sessions. For cortisol however, we can only speculate how these different results originate. One possible explanation pertains to the differences in absolute cortisol increases between both studies. While the stress induction via the Trier Social Stress Test led to a natural cortisol increase, administration of hydrocortisone induced a much larger increase in circulating cortisol. As the MR has a higher affinity for cortisol and is thus already saturated at lower cortisol levels^17^, negative effects of stress have been proposed to be mediated through the glucocorticoid receptor^53^. Thus, it is conceivable that higher cortisol levels due to pharmacological administrations of hydrocortisone are not comparable to the physiological effects of stress. It could be conceivable that cortisol influences interhemispheric information transfer only in a smaller, physiologically plausible range. Another target for cortisol could also be the membrane bound MR receptor^54^. Membrane bound MR is integral to the appraisal process and has been implicated as a resilience factor to psychological disorders^55^. While membrane bound MR has a lower affinity for cortisol than its cytoplasmic counterpart^56^, it mediates rapid cortisol effects under stressful circumstances by altering the excitability of pre- and postsynaptic sites in the limbic system^57^. High levels of circulating cortisol due to pharmacological administration in our study may also exert their effects through the membrane bound MR. It would be interesting for future research to try to differentiate between rapid and delayed effects of cortisol^58,59^.

While there seems to be no influence of acute stress on behavioral FHAs, there is evidence for an effect of stress on neural FHAs^60,61^. In a recent study, our group found changes in frontal alpha asymmetries in response to the Trier Social Stress Test. Moreover, we observed changes in interhemispheric communication in a lexical decision task following stress induction, which indicates, that asymmetric neural processing can be influenced by stress while behavioral asymmetries seem to be rather stable. This is in line with a recent meta-analysis that showed no changes in handedness in depression^62^. Patients with depression regularly display increased cortisol levels indicating a chronic dysregulation of the HPA axis^63^. Moreover, they show changes in frontal alpha asymmetries^64^, thus demonstrating neural changes but not behavioral changes in FHAs.

Robust changes in behavioral and structural asymmetries have mostly been reported for neurodevelopmental disorders like schizophrenia or autism. For example, patients with schizophrenia who suffer from auditory verbal hallucinations display a diminished right ear advantage in response to verbal stimuli and verbal imagery^65^. These disorders have also been associated with changes in cortisol responses and early life stress^12^. It may be therefore reasonable to assume that intrauterine and early life stress could be associated with substantial changes in behavioral and structural asymmetries similarly to a sensitive period. In disorders that typically have a later onset like depression, chronically elevated cortisol levels do not influence already established behavioral asymmetries like handedness but affect asymmetric neural processing. It would be interesting to experimentally test this hypothesis using animal models as has been suggested in a recent opinion paper by Ocklenburg et al.^66^. A recent study in line with these assumptions was conducted by Mundorf et al.^67^, who exposed newborn rats to chronic stress via separation and isolated housing. This chronic stress exposure lead to an increase in asymmetry in turning behavior in rats that were exposed to high levels of stress. Similarly, Somma et al.^68^ found that stress related to a Covid-19 related lock-down increased leftward bias in spatial perception. This indicated that long-term exposure to stress changes behavioral asymmetries.

### Limitations and outlook on future studies

As in our previous study^24^, we only tested women in the follicular phase. This might be a confounding hormonal factor in the female participants as the stage of the menstrual cycle is known to interact with FHAs^8^. As we relied of self-report of cycle phase, it is possible that there are inaccuracies with regard to hormonal status. Future studies should assess hormonal levels in their participants to investigate how cycle phase dependent changes affect cognitive processes^69^. Differential effects may be expected in other cycle phases such as the luteal phase.

It would be interesting to see if long-term administration of cortisol exerts different effects on otherwise stable FHAs. Since these experiments cannot be performed on healthy participants due to ethical concerns, animal models or patient studies might present a possible line of research. For example, patients with Cushing syndrome^70^ or under long-term administration of glucocorticoids^71^ could be studied. Moreover, while behavioral asymmetries are not readily influenced through cortisol administration, it would be valuable to use electroencephalography and functional magnetic resonance imaging to shed light on possible neural changes^60,72^.

### Conclusion

In conclusion, the current study could not show a relationship between cortisol and hemispheric asymmetries after pharmacological hydrocortisone administration. We could also find no evidence for an influence of cortisol on interhemispheric integration. It could be conceivable that cortisol influences interhemispheric information transfer only in a smaller range. A focus on timing and varying doses of cortisol in future research may elucidate this association.

## Supplementary Information

Supplementary Tables.

## References

[1] Ocklenburg S, Güntürkün O (2017). The Lateralized Brain: The Neuroscience and Evolution of Hemispheric Asymmetries.

[2] Knecht S (2000). Language lateralization in healthy right-handers. Brain.

[3] Packheiser J (2020). A large-scale estimate on the relationship between language and motor lateralization. Sci. Rep..

[4] Tzourio-Mazoyer N, Seghier ML (2016). The neural bases of hemispheric specialization. Neuropsychologia.

[5] Conti F, Manzoni T (1994). The neurotransmitters and postsynaptic actions of callosally projecting neurons. Behav. Brain Res..

[6] Ocklenburg S, Ball A, Wolf CC, Genç E, Güntürkün O (2015). Functional cerebral lateralization and interhemispheric interaction in patients with callosal agenesis. Neuropsychology.

[7] Nowicka A, Tacikowski P (2011). Transcallosal transfer of information and functional asymmetry of the human brain. Laterality Asymmetries Body Brain Cogn..

[8] Hausmann M, Güntürkün O (2000). Steroid fluctuations modify functional cerebral asymmetries: The hypothesis of progesterone-mediated interhemispheric decoupling. Neuropsychologia.

[9] Mundorf A, Ocklenburg S (2021). The Clinical Neuroscience of Lateralization.

[10] Wang B (2018). The abnormality of topological asymmetry in hemispheric brain anatomical networks in bipolar disorder. Front. Neurosci..

[11] Carper RA, Treiber JM, DeJesus SY, Müller R-A (2016). Reduced hemispheric asymmetry of white matter microstructure in autism spectrum disorder. J. Am. Acad. Child Adolesc. Psychiatry.

[12] Berretz G, Wolf OT, Güntürkün O, Ocklenburg S (2020). Atypical lateralization in neurodevelopmental and psychiatric disorders: What is the role of stress?. Cortex.

[13] McEwen BS (1998). Protective and damaging effects of stress mediators. N. Engl. J. Med..

[14] Joëls M, Baram TZ (2009). The neuro-symphony of stress. Nat. Rev. Neurosci..

[15] Nater UM, Rohleder N (2009). Salivary alpha-amylase as a non-invasive biomarker for the sympathetic nervous system: Current state of research. Psychoneuroendocrinology.

[16] de Kloet ER, Joëls M, Holsboer F (2005). Stress and the brain: From adaptation to disease. Nat. Rev. Neurosci..

[17] Joëls M, Karst H, DeRijk R, de Kloet ER (2008). The coming out of the brain mineralocorticoid receptor. Trends Neurosci..

[18] Brüne M, Nadolny N, Güntürkün O, Wolf OT (2013). Stress induces a functional asymmetry in an emotional attention task. Cogn. Emot..

[19] Stanković M, Nešić M (2020). Functional brain asymmetry for emotions: Psychological stress-induced reversed hemispheric asymmetry in emotional face perception. Exp. Brain Res..

[20] Gainotti G (2019). Emotions and the right hemisphere: Can new data clarify old models?. Neuroscientist.

[21] Ocklenburg S, Korte SM, Peterburs J, Wolf OT, Güntürkün O (2016). Stress and laterality—The comparative perspective. Physiol. Behav..

[22] Barbaccia ML (1996). Time-dependent changes in rat brain neuroactive steroid concentrations and GABAA receptor function after acute stress. Neuroendocrinology.

[23] Moghaddam B (1993). Stress preferentially increases extraneuronal levels of excitatory amino acids in the prefrontal cortex: Comparison to hippocampus and basal ganglia. J. Neurochem..

[24] Berretz G, Packheiser J, Wolf OT, Ocklenburg S (2020). Dichotic listening performance and interhemispheric integration after stress exposure. Sci. Rep..

[25] Kirschbaum C, Pirke K-M, Hellhammer DH (1993). The ‘Trier Social Stress Test’—A tool for investigating psychobiological stress responses in a laboratory setting. Neuropsychobiology.

[26] Zoccola PM, Dickerson SS, Zaldivar FP (2008). Rumination and cortisol responses to laboratory stressors. Psychosom. Med..

[27] Merz CJ, Hamacher-Dang TC, Stark R, Wolf OT, Hermann A (2018). Neural underpinnings of cortisol effects on fear extinction. Neuropsychopharmacology.

[28] Westerhausen R (2019). A primer on dichotic listening as a paradigm for the assessment of hemispheric asymmetry. Laterality.

[29] Kimura D (1961). Cerebral dominance and the perception of verbal stimuli. Can. J. Psychol. Rev. Can. Psychol..

[30] Prete G, D'Anselmo A, Brancucci A, Tommasi L (2018). Evidence of a right ear advantage in the absence of auditory targets. Sci. Rep..

[31] Prete G, Marzoli D, Brancucci A, Tommasi L (2016). Hearing it right: Evidence of hemispheric lateralization in auditory imagery. Hear. Res..

[32] Westerhausen R, Kompus K (2018). How to get a left-ear advantage: A technical review of assessing brain asymmetry with dichotic listening. Scand. J. Psychol..

[33] Hugdahl K, Westerhausen R (2016). Speech processing asymmetry revealed by dichotic listening and functional brain imaging. Neuropsychologia.

[34] Alzahrani AD, Almuhammadi MA (2013). Left ear advantages in detecting emotional tones using dichotic listening task in an Arabic sample. Laterality Asymmetries Body Brain Cogn..

[35] Belger A, Banich MT (1992). Interhemispheric interaction affected by computational complexity. Neuropsychologia.

[36] Banich MT, Belger A (1990). Interhemispheric interaction: How do the hemispheres divide and conquer a task?. Cortex.

[37] Faul F, Erdfelder E, Lang A-G, Buchner A (2007). G* Power 3: A flexible statistical power analysis program for the social, behavioral, and biomedical sciences. Behav. Res. Methods.

[38] Oldfield RC (1971). The assessment and analysis of handedness: The Edinburgh inventory. Neuropsychologia.

[39] Willems RM, van der Haegen L, Fisher SE, Francks C (2014). On the other hand: Including left-handers in cognitive neuroscience and neurogenetics. Nat. Rev. Neurosci..

[40] Herhaus B, Petrowski K (2018). Cortisol stress reactivity to the trier social stress test in obese adults. Obes. Facts.

[41] Schwabe L, Tegenthoff M, Höffken O, Wolf OT (2010). Concurrent glucocorticoid and noradrenergic activity shifts instrumental behavior from goal-directed to habitual control. J. Neurosci..

[42] Kahan TL, Claudatos S (2016). Phenomenological features of dreams: Results from dream log studies using the Subjective Experiences Rating Scale (SERS). Conscious. Cogn..

[43] Kudielka BM, Schommer NC, Hellhammer DH, Kirschbaum C (2004). Acute HPA axis responses, heart rate, and mood changes to psychosocial stress (TSST) in humans at different times of day. Psychoneuroendocrinology.

[44] Lorentz K, Gütschow B, Renner F (1999). Evaluation of a direct α-amylase assay using 2-chloro-4-nitrophenyl-α-D-maltotrioside. Clin. Chem. Lab. Med..

[45] Ocklenburg S (2013). FOXP2 variation modulates functional hemispheric asymmetries for speech perception. Brain Lang..

[46] Hahn C (2011). Smoking reduces language lateralization: A dichotic listening study with control participants and schizophrenia patients. Brain Cogn..

[47] Arning L (2013). PCSK6 VNTR polymorphism is associated with degree of handedness but not direction of handedness. PLoS ONE.

[48] Pruessner JC, Kirschbaum C, Meinlschmid G, Hellhammer DH (2003). Two formulas for computation of the area under the curve represent measures of total hormone concentration versus time-dependent change. Psychoneuroendocrinology.

[49] Reyes G (2020). Hydrocortisone decreases metacognitive efficiency independent of perceived stress. Sci. Rep..

[50] Langer K, Jentsch VL, Wolf OT (2021). Cortisol promotes the cognitive regulation of high intensive emotions independent of timing. Eur. J. Neurosci..

[51] Kimura D (1967). Functional asymmetry of the brain in dichotic listening. Cortex.

[52] Prete G, Tommasi V, Tommasi L (2020). Right news, good news! The valence hypothesis and hemispheric asymmetries in auditory imagery. Lang. Cogn. Neurosci..

[53] Lupien SJ, Maheu F, Tu M, Fiocco A, Schramek TE (2007). The effects of stress and stress hormones on human cognition: Implications for the field of brain and cognition. Brain Cogn..

[54] Joëls M, de Kloet ER (2017). 30 years of the mineralocorticoid receptor: The brain mineralocorticoid receptor: A saga in three episodes. J. Endocrinol..

[55] ter Heegde F, de Rijk RH, Vinkers CH (2015). The brain mineralocorticoid receptor and stress resilience. Psychoneuroendocrinology.

[56] Karst H (2005). Mineralocorticoid receptors are indispensable for nongenomic modulation of hippocampal glutamate transmission by corticosterone. Proc. Natl. Acad. Sci. U. S. A..

[57] Groeneweg FL, Karst H, de Kloet ER, Joëls M (2011). Rapid non-genomic effects of corticosteroids and their role in the central stress response. J. Endocrinol..

[58] Henckens MJ, van Wingen GA, Joëls M, Fernández G (2010). Time-dependent effects of corticosteroids on human amygdala processing. J. Neurosci..

[59] Henckens MJ, van Wingen GA, Joëls M, Fernández G (2011). Time-dependent corticosteroid modulation of prefrontal working memory processing. Proc. Natl. Acad. Sci. U. S. A..

[60] Quaedflieg CWEM, Meyer T, Smulders FTY, Smeets T (2015). The functional role of individual-alpha based frontal asymmetry in stress responding. Biol. Psychol..

[61] Zhang X (2018). Emotional stress regulation: The role of relative frontal alpha asymmetry in shaping the stress response. Biol. Psychol..

[62] Packheiser J, Schmitz J, Stein CC, Pfeifer LS, Berretz G, Papadatou-Pastou M, Peterburs J, Ocklenburg S (2021). Hair cortisol, stress exposure, and mental health in humans: A systematic review. J. Affect. Disord..

[63] Staufenbiel SM, Penninx BWJH, Spijker AT, Elzinga BM, van Rossum EFC (2013). Hair cortisol, stress exposure, and mental health in humans: A systematic review. Psychoneuroendocrinology.

[64] Thibodeau R, Jorgensen RS, Kim S (2006). Depression, anxiety, and resting frontal EEG asymmetry: A meta-analytic review. J. Abnorm. Psychol..

[65] Altamura M (2020). Do patients with hallucinations imagine speech right?. Neuropsychologia.

[66] Ocklenburg S, Berretz G, Packheiser J, Friedrich P (2020). Laterality 2020: Entering the next decade. Laterality.

[67] Mundorf A, Matsui H, Ocklenburg S, Freund N (2020). Asymmetry of turning behavior in rats is modulated by early life stress. Behav. Brain Res..

[68] Somma F (2021). Further to the left: Stress-induced increase of spatial pseudoneglect during the COVID-19 lockdown. Front. Psychol..

[69] Merz, C. J. & Wolf, O. T. Stress and emotional learning in humans: evidence for sex differences. In *Sex Differences in the Central Nervous System* 149–170 10.1016/B978-0-12-802114-9.00007-X (Elsevier, 2016).

[70] Starkman MN (2013). Neuropsychiatric findings in Cushing syndrome and exogenous glucocorticoid administration. Endocrinol. Metab. Clin..

[71] Coluccia D (2008). Glucocorticoid therapy-induced memory deficits: Acute versus chronic effects. J. Neurosci. Off. J. Soc. Neurosci..

[72] Al-Shargie F (2016). Mental stress assessment using simultaneous measurement of EEG and fNIRS. Biomed. Opt. Express.

