# Supplementary material for: Dichotic listening performance and interhemispheric integration after administration of hydrocortisone
Source: Sci Rep. 2021 Nov 3;11:21581. doi: 10.1038/s41598-021-00896-1 (PMC8566584; doi:10.1038/s41598-021-00896-1)
Supplement: Supplementary file 1 — Supplementary Tables. [file 41598_2021_896_MOESM1_ESM.docx]

Supplement

Gesa Berretz^1*^, Julian Packheiser^1^, Oliver Höffken^2^, Oliver T. Wolf^3^, Sebastian Ocklenburg^1^

^1^: Department of Biopsychology, Institute of Cognitive Neuroscience, Faculty of Psychology, Ruhr University Bochum, Bochum, Germany

^2^: Department of Neurology, BG-University Clinic Bergmannsheil, Ruhr University Bochum, Bochum, Germany.

^3^: Department of Cognitive Psychology, Institute of Cognitive Neuroscience, Faculty of Psychology, Ruhr University Bochum, Bochum, Germany

*Corresponding Author: Gesa Berretz

Address: Ruhr University Bochum Universitätsstraße 150, IB 6-109

Telephone Number: +49 234 32 21453

Fax Number: +49 234 32 14377

E-Mail: Gesa.Berretz@rub.de

|  |  | df | F | p | Partial η² |
| --- | --- | --- | --- | --- | --- |
| Number of correct responses | Treatment | 1 | 2.03 | 0.160 | 0.03 |
|  | Condition | 1 | 512.03 | <0.001 | 0.90 |
|  | Visual field | 1 | 217.78 | <0.001 | 0.79 |
|  | Treatment * Condition | 1 | 0.75 | 0.390 | 0.01 |
|  | Treatment * Visual field | 1 | 0.14 | 0.714 | 0.00 |
|  | Condition * Visual field | 1 | 203.02 | <0.001 | 0.78 |
|  | Treatment * Condition * Visual field | 1 | 0.02 | 0.904 | 0.00 |
| Reaction times | Treatment | 1 | 0.96 | 0.331 | 0.02 |
|  | Condition | 1 | 36.21 | <0.001 | 0.39 |
|  | Visual field | 1 | 0.11 | 0.740 | 0.00 |
|  | Treatment * Condition | 1 | 0.19 | 0.665 | 0.00 |
|  | Treatment * Visual field | 1 | 1.62 | 0.208 | 0.03 |
|  | Condition * Visual field | 1 | 6.73 | 0.012 | 0.11 |
|  | Treatment * Condition * Visual field |  | 0.34 | 0.565 | 0.01 |

Supplementary Table S1. ANOVA of the Banich-Belger task

Supplementary Table S2. ANOVA of verbal dichotic listening task

|  |  | df | F | p | Partial η² |
| --- | --- | --- | --- | --- | --- |
| Number of correct responses | Treatment | 1 | 0.30 | 0.587 | 0.01 |
|  | Ear | 1 | 64.10 | <0.001 | 0.53 |
|  | Treatment * Ear | 1 | 1.23 | 0.273 | 0.02 |
| Reaction times | Treatment | 1 | 0.43 | 0.514 | 0.01 |
|  | Ear | 1 | 20.62 | <0.001 | 0.27 |
|  | Treatment * Ear | 1 | 0.75 | 0.391 | 0.01 |

Supplementary Table S3. ANOVA of emotional dichotic listening task

|  |  | df | F | p | Partial η² |
| --- | --- | --- | --- | --- | --- |
| Number of correct responses | Treatment | 1 | 0.64 | 0.429 | 0.01 |
|  | Ear | 1 | 16.51 | <0.001 | 0.23 |
|  | Treatment * Ear | 1 | 0.47 | 0.497 | 0.01 |
| Reaction times | Treatment | 1 | 0.09 | 0.767 | 0.00 |
|  | Ear | 1 | 21.60 | <0.001 | 0.28 |
|  | Treatment * Ear | 1 | 0.10 | 0.753 | 0.00 |

Supplementary Table S4. Even-odd-reliability coefficients of the Banich-Belger task.

|  | | Treatment | Placebo |
| --- | --- | --- | --- |
| Correlation between Items | | 0.40 | 0.38 |
| Spearman-Brown-Coefficient | even length | 0.58 | 0.55 |
|  | uneven length | 0.58 | 0.55 |
| Guttmans Split-Half-Coefficient | | 0.58 | 0.43 |

Supplementary Table S5. Even-odd-reliability coefficients of the verbal dichotic listening task

|  | | Treatment | Placebo |
| --- | --- | --- | --- |
| Correlation between Items | | 0.96 | 0.97 |
| Spearman-Brown-Coefficient | even length | 0.98 | 0.98 |
|  | uneven length | 0.98 | 0.98 |
| Guttmans Split-Half-Coefficient | | 0.98 | 0.98 |

Supplementary Table S6. Even-odd-reliability coefficients of the emotional dichotic listening task

|  | | Treatment | Placebo |
| --- | --- | --- | --- |
| Correlation between Items | | 0.92 | 0.94 |
| Spearman-Brown-Coefficient | even length | 0.96 | 0.97 |
|  | uneven length | 0.96 | 0.97 |
| Guttmans Split-Half-Coefficient | | 0.96 | 0.97 |
